# Effects of life history stage and climatic conditions on fecal egg counts in plains zebras (*Equus quagga*) in the Serengeti National Park

**DOI:** 10.1007/s00436-020-06836-8

**Published:** 2020-08-11

**Authors:** Peter A. Seeber, Tetiana A. Kuzmina, Alex D. Greenwood, Marion L. East

**Affiliations:** 1 Limnological Institute, University of Konstanz, Constance, Germany; 2 Department of Wildlife Diseases, Leibniz Institute for Zoo and Wildlife Research, Berlin, Germany; 3 Department of Parasitology, I. I. Schmalhausen Institute of Zoology, NAS of Ukraine, Bogdan Khmelnytsky Street, 15, Kyiv, 01030 Ukraine; 4 Department of Veterinary Medicine, Freie Universität Berlin, Berlin, Germany; 5 Department of Evolutionary Dynamics, Leibniz Institute for Zoo and Wildlife Research, Berlin, Germany

**Keywords:** Strongylidae, Ascarididae, Anoplocephalidae, Parasite burden, Parasite prevalence

## Abstract

In wildlife, endoparasite burden can be affected by host life history stage, environmental conditions, host abundance, and parasite co-infections. We tested the effects of these factors on gastrointestinal parasite infection in plains zebras (*Equus quagga*) in the Serengeti ecosystem, Tanzania, using fecal egg counts of two nematode families (Strongylidae and Ascarididae) and the presence/absence of cestode (Anoplocephalidae) eggs. We predicted higher egg counts of Strongylidae and Ascarididae, and increased likelihood of Anoplocephalidae infection in individuals (1) during energetically costly life history stages when resource allocation to immune processes may decrease and in young zebras after weaning because of increased uptake of infective stages with forage, (2) when climatic conditions facilitate survival of infective stages, (3) when large zebra aggregations increase forage contamination with infective stages, and (4) in individuals co-infected with more than one parasite group as this may indicate reduced immune competence. Strongylidae egg counts were higher, and the occurrence of Anoplocephalidae eggs was more likely in bachelors than in band stallions, whereas Ascarididae egg counts were higher in band stallions. Strongylidae and Ascarididae egg counts were not increased in lactating females. Strongylidae egg counts were higher in subadults than in foals. Regardless of sex and age, Ascarididae infections were more likely under wet conditions. Co-infections did not affect Strongylidae egg counts. Ascarididae egg counts in adult females were higher when individuals were co-infected with Anoplocephalidae. We present evidence that parasite burdens in plains zebras are affected by life history stage, environmental conditions, and co-infection.

**Electronic supplementary material:**

The online version of this article (10.1007/s00436-020-06836-8) contains supplementary material, which is available to authorized users.

## Introduction

The mammalian intestinal tract is inhabited by a diverse and dynamic parasite community that is shaped throughout the host’s lifespan by interactions between various internal and external factors (Krecek et al. 1987a; Behnke et al. 2005; Graham 2008; Telfer et al. 2010; Hayward et al. 2011; Van der Wal et al. 2014; Kappeler et al. 2015). Unraveling the factors driving parasite community dynamics in wild mammals at the individual and population level is difficult (Pedersen and Fenton 2007) and is hampered by insufficient knowledge of the parasite species involved and their biology. There is also a current lack of non-invasive (fecal) assays for wild mammalian species to measure relevant immunological responses to gastrointestinal parasite infections and how these responses are changed by prevailing conditions such as season, nutrition, and physiological stress throughout an individual’s lifespan (Lee 2006; Martin et al. 2008; Ardia et al. 2011). Even so, previous studies revealed substantial variation between individuals in terms of their burden of infection with various parasite taxa, the diversity of co-infecting parasite taxa, and how these measures change over time (Irvine et al. 2000; Behnke et al. 2005; Irvine 2006; Ferreira et al. 2019). Individuals differ in their exposure to parasite infective stages, in their ability to mount immune responses to control or clear infections due to factors such as climatic conditions, age, and diet (Cattadori et al. 2005; Behnke et al. 2005; Råberg et al. 2009; Turner and Getz 2010; Hayward et al. 2011; Ferreira et al. 2019).

Life history theory predicts that when food intake is insufficient to sustain all life processes during energetically costly life history stages, allocation of resources to immune processes may be reduced (Stearns 1992; Roff 1993), leading to increased susceptibility to infection. Increased parasite burdens during energetically costly life stages such as lactation (Festa-Bianchet 1989; Malan et al. 1997; East et al. 2015) or during periods when males compete for mate competition (Decristophoris et al. 2007; Corlatti et al. 2012; Ezenwa et al. 2012) may lead to reduced allocation of resources to immune processes. Furthermore, infection with energetically costly parasites may drain body resources, thereby undermining a host’s ability to mount effective immune responses to current or subsequent infections, thereby facilitating persistence of existing parasite infections and increasing susceptibility to co-infections (Beldomenico et al. 2008; Beldomenico and Begon 2010; Jolles et al. 2015; Mabbott 2018). In mammals, immunological resistance to infections changes with age, being generally lower in young animals than adults (Dowling and Levy 2014; Simon et al. 2015; Beirne et al. 2016), suggesting that parasites that are controlled by adaptive immune responses may be more prevalent during early life stages.

We investigated factors shaping gastrointestinal parasite infection burdens and the occurrence of co-infections in plains zebras (*Equus quagga* Boddaert, 1785) in the Serengeti National Park, Tanzania, focusing on parasite taxa that are known to infect plains zebras in Africa and that rely on ingestion of infective stages for transmission, i.e., nematodes of the families Strongylidae and Ascarididae and cestodes of the family Anoplocephalidae (Krecek et al. 1987b; Wambwa et al. 2004; Turner and Getz 2010; Fugazzola and Stancampiano 2012; Cizauskas et al. 2015). Egg counts of nematodes were considered a proxy indicator of infection burden, and presence or absence of cestode eggs provided a binary index of cestode infection.

The biology of the three parasite families considered in this study provides insights into the likely energetic cost of infection and factors likely to influence the survival and transmission of infective stages and the period of time required for the development adult worms following infection. Strongylidae nematodes have a direct life cycle in which adult females shed eggs into the lumen of the host’s large intestines that are then shed into the environment with feces. After larval development, infective larvae migrate away from host feces onto grass, and infection occurs when infective larvae are ingested with forage (Duncan 1974; Nielsen and Reinemeyer 2018). Following ingestion, larvae of large strongyles (subfamily Strongylinae) typically migrate through various host tissues, causing energetically costly tissue damage and repair, before they move into the lumen of the large intestine where they develop into adult worms. Larvae of small strongyles (subfamily Cyathostominae) either directly develop into adult worms (without long tissue migration) or encyst in the host’s intestinal mucosa of the large intestines where, in domestic horses, they may remain for typically 3–4 months (Corning 2009). Cyathostomins larvae are more likely to encyst when high numbers of adult cyathostomins occur in the host’s large intestine (Corning 2009), and mass penetration of the intestinal mucosa and mass emergence of encysted larvae into the gut lumen may have energetically costly pathogenic effects on hosts. The speed of Strongylidae larval development and survival in the environment are affected by climatic factors, particularly temperature and humidity (Nielsen et al. 2007). Low moisture content in host feces and herbage is a major constraint for larval development and transmission as movement onto surrounding herbage, and subsequent transmission is impaired in dry environments (Berbigier et al. 1990; Stromberg 1997). In general, this predicts higher strongyle infection burden in plains zebras during wet than during dry climatic conditions.

Two nematode species from the family Ascarididae are known to infect equids—*Parascaris equorum* and *P. univalens* (Nielsen et al. 2014). Ascarids have a direct life cycle, with adult worms inhabiting the host’s small intestines and females shedding eggs into the intestinal lumen which enter the environment with the host’s feces (Bowman 2013). Infective second-stage larvae develop in eggs within approximately 10–14 days at 25–35 °C (Clayton 1986). Larvated eggs can survive in the environment for up to 10 years (Reinemeyer 2009), and infection of susceptible hosts occurs when larvated eggs are ingested with food or water (Bowman 2013). Ascarididae infections in domestic horses induce acquired immunity typically during the first year of life; hence, parascarid infections are infrequent in horses older than 2 years (Reinemeyer 2009). In plains zebras, parascarid infection should predominately occur in subadults rather than foals because subadults are more likely to ingest ascarid contaminated forage or water than foals that are nursed more often than subadults (Seeber et al. 2019). It is not known whether repeated ascarid infection is necessary to maintain immunity, but even so, contamination of forage and water is expected to be sufficient to maintain a relatively high level of herd immunity. The peak foaling period of plains zebras in the Serengeti ecosystem occurs during the rainy season (Klingel 1969a) which boosts the number of zebras susceptible to ascarid infection.

Three species of cestodes from the family Anoplocephalidae infect equids, *Anoplocephala perfoliata*, *A. magna*, and *Paranoplocephala mamillana* (Lichtenfels 1975; Lyons et al. 2006; Nielsen 2016), and Anoplocephalidae are known to infect plains zebra in Africa (Wambwa et al. 2004; Turner and Getz 2010). Eggs of Anoplocephalidae cestodes are shed in the host’s feces must be ingested by intermediate hosts, which are oribatid mites, and cysticercoid larvae hatch and develop within the mites. Cysticercoids become infective around 2 weeks after hatching, and equids become infected when they ingest forage contaminated with infected mites (Lyons et al. 2006; Roczen-Karczmarz and Tomczuk 2016). Cysteroids attach to the mucosa in the ileum, at the ileocaecal valve, and in the cecum, and after approximately 6–10 weeks, they transform into adults (Lyons et al. 2006). In domestic horses, infections rarely cause clinical symptoms (Bowman 2013), but high infection burdens may cause ulceration and intestinal perforation, particularly in young individuals (Lyons et al. 2006; Nielsen 2016; Nielsen and Reinemeyer 2018). Oribatid mites are most numerous on pasture during warm humid conditions which also favor the survival of cestode eggs (Denegri and de Alzuet 1992; Lyons et al. 2006). We expected ingestion of cysticercoid-infected mites to mostly occur when wet climatic conditions prevail, and adult female worms to start producing eggs approximately 8–12 weeks later; however, adult worms can shed eggs for 4–6 months (Lyons et al. 2006), which may dilute climatic effects on the occurrence of cestode infection.

The majority of the approximately 200,000 plains zebras in the Serengeti ecosystem (IUCN 2017) undertake a long-distance migration at the beginning of the rainy season (in approximately November) from their dry-season range in the north of the ecosystem to the short grass plains in the south where they occur in large aggregations until the end of the rainy season (approximately in May) when they migrate back to their dry-season range (Klingel 1969b). During the dry season, plains zebras generally graze in smaller aggregations spread over larger areas than when on the short grass plains in the rainy season (Maddock 1979). Areas with large aggregations of plains zebras should have greater fecal contamination of forage, hence more infective parasite stages, than areas with smaller aggregations. Rapid regrowth of grazed areas during the rainy season permits repeated grazing of areas when climatic conditions are favorable for the survival of parasite eggs and infective larvae. This suggests that contamination of forage with infective stages is probably higher during wet than during dry climatic conditions, particularly in areas that are repeatedly grazed by large aggregations of plains zebra. When in large aggregation, plains zebras have higher fecal glucocorticoid metabolite concentrations than those in smaller aggregation (Seeber et al. 2018) which may compromise immune function and increase susceptibility to parasite infection (Munck et al. 1984; Hofer and East 2012). Plains zebras need to drink regularly (Cain et al. 2012); thus, fecal contamination of water sources may also contribute to the transmission of nematode infections, particularly when dry weather conditions prevail, and plains zebras visit drinking sites in large aggregations. Together, these factors predict high transmission rates of infectious stages when plains zebra form large aggregations, leading to elevated infection burdens with energetically costly nematode parasites and an increased likelihood of co-infection with cestodes. In plains zebra, because foals are nursed more often than subadults (Seeber et al. 2019), they are less likely to ingest forage or water contaminated with infective larvae of the three parasite types considered. For this reason, foals should have lower nematode infection burdens and a lower probability of cestode infection than subadults.

In summary, our expectations (that are not mutually exclusive) were that parasite burdens should increase (1) during life history stages that are energetically costly or entail a dietary transition from nursing to grazing, (2) during wet climatic conditions which facilitate faster development and increased survival rates of infective stages of all three parasite types (and of the intermediate hosts of cestodes), (3) during times of large aggregations when contamination of forage or water with parasite infective stages increases, and (4) in individuals that are infected with more than one of the parasite groups in question, as co-infections may indicate higher susceptibility (Mabbott 2018).

## Materials and methods

### Sample collection

Field work was carried out in the Serengeti National Park in northern Tanzania during January–March, June–July, and September–October 2016. A total of 253 fresh fecal samples was opportunistically collected from animals within approximately 150 m from the research vehicle. Several fecal boli were sampled from each individual and were then pooled, thoroughly mixed, and fixed in 4% formalin until egg counts were performed (Cossío-Bayúgar et al. 2015). Samples were collected in two types of habitat: (1) the short grass plains in the south part of the park (January–March), where zebras typically aggregate during the rainy season, and (2) open woodland savanna with longer and coarser grass species in the center of the park, here termed woodland boundary (June–July and September–October), where zebras occurred during the dry season. In 2016, the Serengeti ecosystem experienced an El Niño climatic event which caused above-average rainfall and an extension of precipitation into the typical dry season. As a proxy for seasonal wet and dry climatic conditions and as an index of humidity and moisture content in the herbaceous layer, the proportion of green to dry herbaceous vegetation at sampling locations was visually scored using two categories (modified after Shrader et al. 2006), i.e., green (≥ 90% green) or brown (aging/senescent vegetation that is less than 90% green). We categorized zebra aggregation size for each sampled animal by estimating the number of individuals within approximately 500 m in all directions of the research vehicle, using the categories “small aggregation” (≤ 200 zebras) or “large aggregation” (> 200 zebras). In total, 117 fecal samples were collected from zebras in large and 136 samples from zebras in small aggregations.

For each sample, we recorded the individual’s age class, and in adult zebras, their reproductive state. Age class was estimated by total body size (withers height) following Klingel (1969b) and classified as one of the three categories: adult, subadult, and foal (Seeber et al. 2018). Adult males were categorized as band stallions if they were the only adult male in a family group consisting of females and young, or as bachelor males if they were in all-male groups. The reproductive state of adult females was classified as either “lactating” (mares with a suckling foal at foot) or “not lactating.” Thus, sampled zebras were categorized using six life history stages according to age, sex, and reproductive state: (1) foals (*N* = 18), (2) subadults (*N* = 26), (3) bachelor adult males (*N* = 39), (4) band stallions (*N* = 46), (5) non-lactating adult females (*N* = 86), and (6) lactating adult females (*N* = 38).

### Fecal egg counts

In total, 253 fecal samples (from 209 adult and 44 young zebras, i.e., foals and subadults) were examined. Parasite egg counts were conducted according to a modified McMaster flotation protocol (Gordon and Whitlock 1939). Two gram feces was added to 28 mL saturated NaCl solution which was then thoroughly mixed and passed through a strainer to remove debris. McMaster chambers with a volume of 0.15 mL were loaded using a pipette, and parasite eggs were counted after 5 min using a light microscope with 100-fold magnification. Four McMaster chambers were counted per sample to produce an average value. Count data are presented as number of eggs per gram feces (EPG). Eggs were identified by morphology (Bowman 2013) to distinguish three parasite groups: (1) Strongylidae type (family Strongylidae) which included large and small strongylids; (2) Ascarididae type (family Ascarididae) which included eggs of *Parascaris equorum* and, possibly, *P. univalens*, and (3) Anoplocephalidae type (family Anoplocephalidae) which included eggs of *Anoplocephala perfoliata* and, possibly, *A. magna* and *Anoplocephaloides mamillana*. Eggs of Anoplocephalidae can be released while contained within a tapeworm segment which can result in an irregular and clumped distribution; therefore, fecal egg counts may not have any relationship to infection burden (Slocombe 1979; Lyons et al. 2006; Nielsen 2016). Cestode infection was scored as either “infected” (when any number of Anoplocephalidae eggs was recorded) or “non-infected” when no eggs were detected.

### Statistical analyses

Statistical analyses were performed using R software version 3.5.2 (R Development Core Team 2016). Egg counts of Strongylidae and Ascarididae, respectively, were analyzed separately for adult male, adult female, and young zebras (i.e., foals and subadults). For Strongylidae egg counts, we fitted generalized linear models (GLM) with a negative binomial distribution to account for over-dispersed data distribution (Sebatjane et al. 2019) using the R package MASS version 7.3-51.4 (Venables and Ripley 2002), and we used the predictors climatic condition, reproductive state (in adults), age class (in young animals), aggregation size, and binary infection scores (infected or not infected) for each Anoplocephalidae and Ascaridae co-infection.

Numerous fecal samples produced zero Ascaridae egg counts (Table 1); hence for Ascaridae egg counts, we fitted GLMs with a zero-inflated negative binomial (ZINB) distribution, which showed superior fit over models with non-zero-inflated negative binomial distribution; goodness of fit was tested using a likelihood ratio test for model selection with the R package pscl (Jackman 2020). ZINB models involve two steps: a binary model which tests the probability of the dependent variable being zero and a count model which tests how the respective predictor affects the dependent variable if it is not zero. ZINB models applied to Ascaridae egg count data included the predictors climatic condition, reproductive state (or age class, in young animals), aggregation size, and a binary infection score for Anoplocephalidae co-infection. To test the occurrence of Anoplocephalidae infection, we fitted binary logistic regression models with the predictors climatic condition, reproductive state (in adults), age class (in young), aggregation size, and co-infections with Ascarididae (presence/absence of eggs). A summary of the statistical analyses is provided in Supplementary Table 1.

**Table 1:** Prevalence ([prev]; percent of infected individuals), mean number of eggs per gram feces (EPG), mean egg counts including non-infected individuals, and median egg counts of Strongylidae and Ascarididae and prevalence of Anoplocephalidae infections in adult males, adult females, subadults, and foals

	Strongylidae				Ascarididae				Anoplo-cephalidae
	Prev	Mean EPG of infected zebras	Mean EPG including non-infected zebras	Median EPG	Prev	Mean EPG of infected zebras	Mean EPG including non-infected zebras	Median EPG	Prev
Adult males (*N* = 85)	100%	1341	1341	1100	33%	168	55	0	24%
Adult females (*N* = 124)	98%	1172	1153	1175	30%	138	41	0	12%
Subadults (*N* = 26)	100%	1987	1987	1838	34%	314	109	0	23%
Foals (*N* = 18)	88%	971	850	512	25%	175	44	0	13%

Considering the large size and continuously mobile nature of the zebra population and that the locations in which feces were collected were frequently changed during each day, we consider the chance of pseudo-replication due to repeated sampling of the same animal to be negligible. The chance of sampling the same animal in both the wet and dry season ranges is also considered to be small given the diverse migration routes used by plains zebra to transit from the wet to the dry season range. The distribution of samples in wet and dry climatic conditions and in small and large aggregations is shown in Supplementary Table 2. Statistical significance is reported at *p* ≤ 0.05.

## Results

### Prevalence and egg counts

Strongylidae eggs were observed in 98% of 253 fecal samples (99% of adult zebras and 95% of young). In infected zebras, egg counts ranged from 100 to 4475 EPG in adult zebras and from 175 to 4350 EPG in young zebras. Ascarididae eggs were observed in 31% of all samples (adult zebras 32%; young zebras 31%), with egg counts in infected individuals ranging from 25 to 675 EPG in adults and from 25 to 1625 EPG in young zebras. Anoplocephalidae eggs were observed in 18% of all samples (17% of adult zebras and 19% of young). The prevalence of each parasite type and mean egg counts in each sex/age category are shown in Table 1. The frequencies of Strongylidae and Ascarididae egg counts in adult males, adult females, and young were over-dispersed (Supplementary Fig. 1).

### Effect of life history stage

Bachelor stallions had significantly higher Strongylidae egg counts (*z* = − 2.48, *p* = 0.013; Table 2; Fig. 2) and were more frequently infected with Anoplocephalidae (*z* = − 2.38, *p* = 0.017; Table 4; Fig. 3) than band stallions, whereas Ascarididae egg counts were higher in band stallions (*z* = 2.08, *p* = 0.037; Table 3), and there was a non-significant trend for ascarid egg counts to increase with aggregation size (*z* = 1.90, *p* = 0.057; Table 3). In mares, reproductive state had no significant effect on Strongylidae and Ascarididae egg counts or on Anoplocephalidae occurrence (Tables 2, 3, and 4). Subadult zebras had higher Strongylidae egg counts than foals (*z* = 3.37, *p* < 0.001; Table 2), whereas no significant effect of age class on Ascarididae egg counts (Table 3) or on the occurrence of Anoplocephalidae infection (Table 4) was observed.

**Table 2:** Results of generalized linear models on Strongylidae egg counts to test effects of climatic condition, reproductive state (in adults), age class (in young), and co-infection with Ascarididae or Anoplocephalidae in adult males, females, and young plains zebras

Predictor	Direction of the effect	Est.	*z* value	*P*
Adult males				
Intercept		7.49	53.57	< 0.001
Climatic condition	Dry → wet	− 0.13	− 0.85	0.393
Reproductive state	Bachelor → band stallion	*− 0.30*	*− 2.48*	*0.013*
Aggregation size	Small → large	0.14	− 1.01	0.317
Co-infection with Ascarididae	Non-infected → infected	− 0.05	− 0.37	0.713
Co-infection with Anoplocephalidae	Non-infected → infected	− 0.03	− 0.17	0.862
Adult females				
Intercept		7.09	43.55	< 0.001
Climatic condition	Dry → wet	− 0.10	− 0.65	0.519
Reproductive state	Non-lactating → lactating	− 0.09	− 0.62	0.538
Aggregation size	Small → large	> 0.001	0.01	0.996
Co-infection with Ascarididae	Non-infected → infected	0.20	1.34	0.180
Co-infection with Anoplocephalidae	Non-infected → infected	− 0.14	− 0.69	0.490
Young				
Intercept		7.51	25.67	< 0.001
Climatic condition	Dry → wet	0.02	0.06	0.952
Age class	Foal → subadult	*1.04*	*3.37*	*< 0.001*
Aggregation size	Small → large	0.08	0.25	0.805
Co-infection with Ascarididae	Non-infected → infected	0.61	1.07	0.088
Co-infection with Anoplocephalidae	Non-infected → infected	− 0.18	− 0.46	0.643

Significant effects in italics; arrows indicate the direction of the respective effect:; *est.* estimate

**Table 3:** Results of zero-inflated negative binomial models (binary model [indicating the probability of the variable being zero] and count model output) on Ascarididae egg counts to test effects of climatic condition, reproductive state (in adults), age class (in young), and Anoplocephalidae co-infection in adult males, females, and young plains zebras

	Direction of effect	Binary model			Count model		
		Est.	*z* value	*p*	Est.	*z* value	*p*
Adult males							
Intercept		2.11	2.92	0.004	5.41	14.68	< 0.001
Climatic condition	Dry → wet	*− 2.23*	*− 3.12*	*0.002*	− 0.56	− 1.68	0.094
Reproductive state	Bachelor → band stallion	− 0.16	− 0.32	0.751	*0.56*	*2.08*	*0.037*
Aggregation size	Small → large	0.88	1.26	0.207	0.65	1.90	0.057
Co-infection with Anoplocephalidae	Non-infected → infected	0.02	0.04	0.975	− 0.29	− 0.98	0.326
Adult females							
Intercept		2.24	3.38	< 0.001	4.65	9.77	< 0.001
Climatic condition	Dry → wet	*− 1.72*	*− 3.25*	*0.001*	− 0.13	− 0.39	0.700
Reproductive state	Non-lactating → lactating	0.18	0.38	0.706	− 0.21	− 0.66	0.509
Aggregation size	Small → large	0.66	1.33	0.185	− 0.01	− 0.04	0.968
Co-infection with Anoplocephalidae	Non-infected → infected	*− 1.76*	*− 2.84*	*0.005*	*0.51*	*1.96*	*0.050*
Young							
Intercept		2.50	2.60	0.009	6.44	12.21	< 0.001
Climatic condition	Dry → wet	*− 2.48*	*− 2.49*	*0.013*	0.21	0.50	0.617
Age class	Foal → subadult	− 0.67	− 0.81	0.416	0.62	1.57	0.117
Aggregation size	Small → large	1.58	1.66	0.097	*0.99*	*2.40*	*0.016*
Co-infection with Anoplocephalidae	Non-infected → infected	− 1.45	− 1.51	0.131	*− 1.44*	*− 3.03*	*0.003*

Significant effects in italics; arrows indicate the direction of the respective effect *est.* estimate

**Table 4:** Results of models on presence/absence of Anoplocephalidae eggs to test effects of climatic condition, reproductive state (in adults), age class (in young), and co-infection with Ascarididae in adult males, females, and young plains zebras

Predictor	Direction of the effect	Est.	*z* value	*p*
Adult males				
Intercept		1.20	11.99	< 0.001
Climatic condition	Dry → wet	*0.26*	*2.20*	*0.028*
Reproductive state	Bachelor → band stallion	*− 0.21*	*− 2.38*	*0.017*
Aggregation size	Small → large	− 0.12	− 1.26	0.207
Co-infection with Ascarididae	Non-infected → infected	− 0.01	− 0.10	0.917
Adult females				
Intercept		1.07	14.30	< 0.001
Climatic condition	Dry → wet	0.03	0.36	0.721
Reproductive state	Non-lactating → lactating	− 0.01	− 0.29	0.772
Aggregation size	Small → large	0.05	0.84	0.400
Co-infection with Ascarididae	Non-infected → infected	*0.21*	*3.24*	*0.001*
Young				
Intercept		1.27	11.81	< 0.001
Climatic condition	Dry → wet	− 0.13	− 0.83	0.409
Age class	Foal → subadult	0.06	0.50	0.619
Aggregation size	Small → large	0.17	1.40	0.162
Co-infection with Ascarididae	Non-infected → infected	0.22	1.61	0.107

Significant effects in italics; arrows indicate the direction of the respective effect: *est.* estimate

### Effects of climatic conditions and aggregation size

**Fig. 1:**
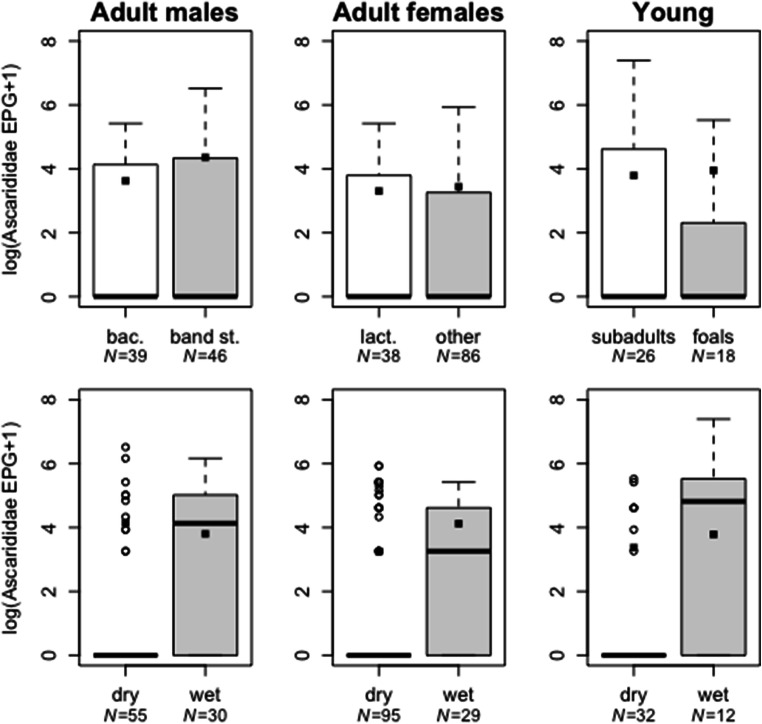
Ascarididae egg counts (EPG; [log+1]-transformed) in adult male, adult female, and young zebras at different life history stages (adult males: bachelors [bac.] and band stallions [band st.]; adult females: lactating [lact.] and non-lactating [other]; young: subadults and foals) and under different climatic conditions (dry vs. wet). Boxes indicate first and third quartiles; center lines indicate median values; whiskers extend to the highest (and lowest) value within 1.5 times the inter-quartile range. Data points beyond the end of the whiskers are plotted as open dots. Filled squares indicate the mean predicted effect of the respective predictor according to the zero-inflated count models

No effect of season on Strongylidae (Table 2) and Ascarididae egg counts (Table 3, count models) was observed in adult males, adult females, and young zebras. The binary stage of the ZINB models indicated that Ascarididae egg counts were less likely to be zero under wet than under dry conditions in adult males, adult females, and young zebras (*z* = − 3.12, *p* = 0.002; *z* = − 3.25, *p* = 0.001; *z* = − 2.49, *p* = 0.013, respectively; Table 3, binary models; Fig. 1). Anoplocephalidae infection was more frequent during wet than during dry conditions in stallions (*z* = 2.20, *p* = 0.028; Fig. 3) but was not affected by season in adult females or young zebras (Table 4). Aggregation size produced no effect on Strongylidae and Ascarididae egg counts and on Anoplocephalidae infection frequency in adult males and females (Tables 2, 3, and 4). In young zebras, Ascarididae egg counts were higher in large than in small aggregations (*z* = 2.4, *p* = 0.016; Table 3, count model, Fig. 1).

### Effect of co-infections

Strongylidae egg counts were not affected by co-infection with Ascarididae or Anoplocephalidae in adult males, adult females, or young zebras (Table 2). Adult females were more likely to be infected with Ascarididae (*z* = − 2.84, *p* = 0.005; Table 3), and these egg counts were significantly higher when they were also infected with Anoplocephalidae (*z* = 1.96, *p* = 0.05; Table 3), whereas in young, Ascarididae egg counts were higher in individuals without Anoplocephalidae infection (*z* = − 3.03, *p* = 0.003; Table 3). The presence of Anoplocephalidae infection was not affected by co-infection with Ascarididae in adult males and young but was more likely in females when they were infected with Ascarididae (*z* = 3.24, *p* = 0.001; Table 4).

## Discussion

Our study on gastrointestinal parasite infection in the large migratory population of plains zebras in the Serengeti National Park revealed considerable individual heterogeneity in Strongylidae and Ascarididae egg counts (Table 1, Supplementary Fig. S1) as reported by previous studies on equids in African and other ecosystems (Scialdo-Krecek et al. 1982; Krecek et al. 1987b, a, 1989; Matthee et al. 2004; Kuzmina et al. 2007, 2013; Kornaś et al. 2010; Getachew et al. 2010; Fugazzola and Stancampiano 2012). Our analyses revealed evidence that both nematode infection burdens and the occurrence of cestode infection were affected by life history stage and climatic factors (Tables 2, 3, and 4).

### Life history stage

**Fig. 2:**
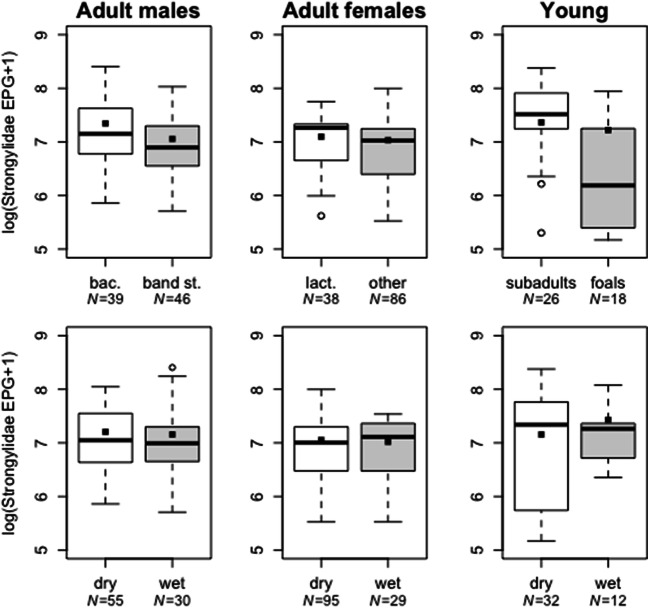
Strongylidae egg counts (EPG; [log+1]-transformed) in adult male, adult female, and young zebras at different life history stages (adult males: bachelors [bac.] and band stallions [band st.]; adult females: lactating [lact.] and non-lactating [other]; young: subadults and foals) and under different climatic conditions (dry vs. wet). Boxes indicate first and third quartiles; center lines indicate median values; whiskers extend to the highest (and lowest) value within 1.5 times the inter-quartile range. Data points beyond the end of the whiskers are plotted as open dots. Filled squares indicate the mean predicted effect of the respective predictor according to the zero-inflated count models

**Fig. 3:**
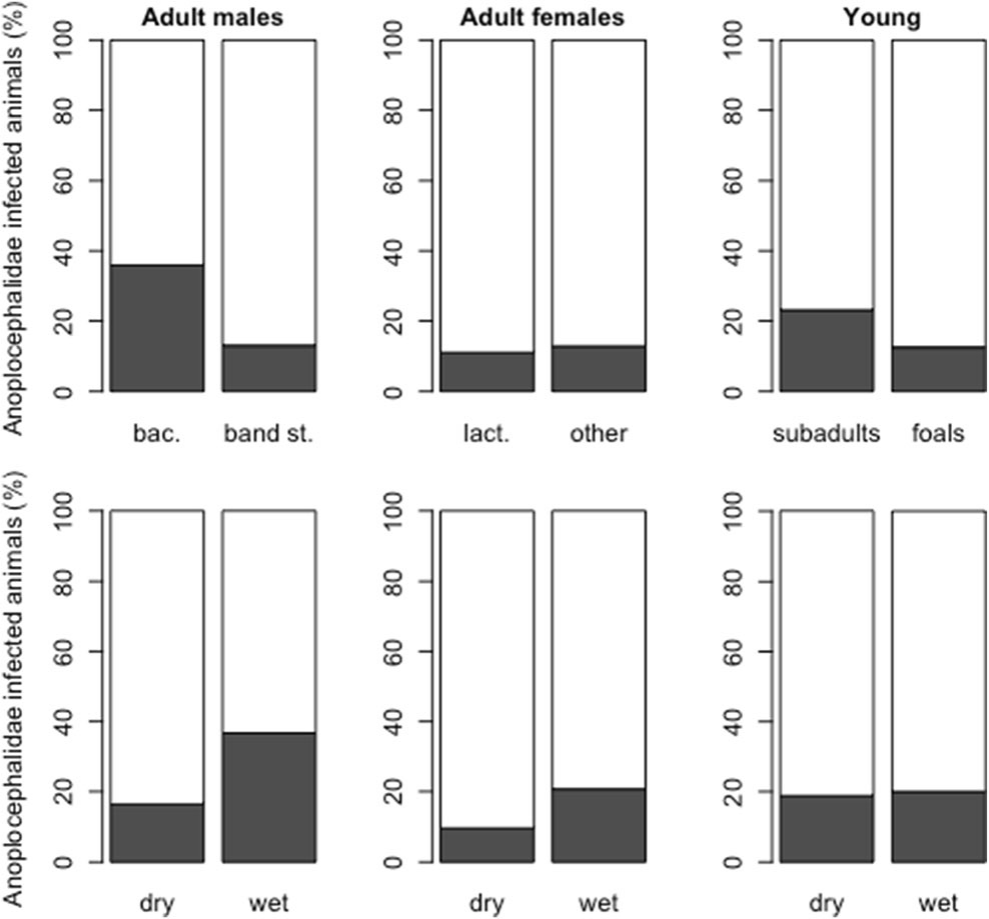
Percentage of adult male, adult female, and young zebras infected (gray) and not infected with Anoplocephalidae (white) at different life history stages (adult males: bachelors [bac.] and band stallions [band st.]; adult females: lactating [lact] and non-lactating [other]; young: subadults and foals) and under different climatic conditions (dry vs. wet)

The occurrence and intensity of parasite infections can increase during energetically costly life history stages when insufficient food intake leads to reduced allocation of resources to immune processes (Stearns 1992). In mammals, sex differences in immune responses render males more prone to pathogen infection than females (Klein and Flanagan 2016). Our findings revealed that band stallions had higher Ascarididae egg counts than bachelor males (Table 3, Fig. 1), whereas bachelor males had higher Strongylidae egg counts (Table 2, Fig. 2) and were more likely to be co-infected with Anoplocephalidae than band stallions (Table 4, Fig. 3). Band stallions were previously found to have higher fecal glucocorticoid metabolite concentrations than bachelor males (Seeber et al. 2018), and band stallions likely have higher androgen concentrations than bachelor males, as is the case for breeding stallions in domestic horses (McDonnell and Murray 1995). In some other mammals, male social dominance is associated with elevated glucocorticoid and androgen concentrations, as well as increased parasite burden (Muehlenbein 2006; Corlatti et al. 2012). The effects of both glucocorticoids and androgens on immune functions and parasite infections may be complex. For example, in male Grant’s gazelle (*Nanger granti*), testosterone is negatively associated with measures of adaptive immunity and positively associated with measures of innate immunity (Ezenwa et al. 2012). It is plausible that in our study, elevated glucocorticoid and androgen levels in band stallions reduced acquired immune responses and compromised the ability of band stallions to control Ascarididae infections. However, the observed higher Strongylidae egg counts in bachelor plain zebra males contrast those of a positive association between testosterone and Strongylidae egg counts in male Alpine ibex (*Capra ibex*) (Decristophoris et al. 2007) and the lack of an effect of testosterone on Strongylidae egg counts in Grant’s gazelles (Ezenwa et al. 2012).

In adult females, we found no evidence that lactation affected nematode and ascarid egg counts or cestode infections (Tables 2, 3, and 4). These findings suggest that lactating zebra mares in the Serengeti ecosystem resort to resource allocation trade-offs that do not compromise their immune processes, possibly because they increase their time spent foraging (Seeber et al. 2019). This suggestion is supported by lactating females not having higher fecal glucocortid metabolite concentrations than other adult females (Seeber et al. 2018). In contrast to our findings, lactating female plains zebra in the more highly seasonal environment of Etosha National Park, Namibia, were reported to have elevated strongyle egg counts (Cizauskas et al. 2015). Overall, our results from adult plain zebra indicate that the interplay of the host’s endocrine system, immune system, and intestinal parasite burden is complex.

The transition of zebra foals from nursing to grazing was predicted to increase uptake of infective parasite stages with forage; thus, nursing young should have lower infection burdens than subadults. In line with this prediction, we found higher strongylid egg counts in subadult zebras than foals (Table 2, Fig. 2), probably because subadults spend more time grazing than foals (Seeber et al. 2019) which increases the likelihood of ingesting infective stages of parasites (Duncan 1974; Kornaś et al. 2010; Kuzmina et al. 2016). Similarly, in domestic horses, yearlings had higher numbers of Strongylidae than foals (Boersema et al. 1996).

### Climatic conditions and aggregation

Climatic conditions such as humidity affect the development and survival of parasite eggs and infective stages and the likelihood of transmission (Duncan 1974; Mfitilodze and Hutchinson 1988; Stromberg 1997; Kuzmina et al. 2006; Turner and Getz 2010; Leathwick et al. 2015; Cizauskas et al. 2015). We predicted greater survival of infective stages under wet rather than under dry conditions. In line with this prediction, we found that regardless of age or reproductive category, plains zebras were more likely to be infected with ascarids in months when the prevailing weather conditions were wet rather than dry, even though egg counts were not affected by climatic conditions (Table 3, Fig. 1). However, given that reproduction in these nematodes starts 3 to 4 months after infection (Reinemeyer 2009; Bowman 2013; Nielsen and Reinemeyer 2018), our results suggest that some of the infected animals may have acquired infection during the previous dry season. Adaptive immune responses following infection in domestic horses result in the clearance of infection in most animals by 2 years of age, but our results do not fully fit this pattern, as infection prevalence was approximately 30% in the adult population (Table 1). Presumably, zebras infected during the previous dry season would clear infections once they developed an adequate adaptive immune response. Hence, zebras infected in the previous dry season should cease to be susceptible to further infection at some point during the wet season when they develop immunity.

In the Serengeti ecosystem, most plains zebra are born between January and March (Klingel 1969a), which leads to a spike in the number of susceptible young animals during the rainy season. The development of immunity against ascarid infection following infection in young zebras increases herd immunity and reduces the number of susceptible animals in the population. The ingestion of ascarid eggs by immune plains zebra and by other herbivores such as the approximately 1 million strong wildebeest population (which follows the same migratory pattern as plains zebra) individuals may reduce environmental contamination with ascarid eggs to some extent, thereby acting as an encounter-reduction/dilution effect (Keesing et al. 2006). We found evidence of greater likelihood of infection with Anoplocephalidae cestodes in adult males during wet than dry conditions, but this was not the case for adult females or young. We suggest that physiological and behavioral changes in males during the peak breeding period, which occurs in the wet season (Klingel 1969a), may explain these findings.

We found no evidence that weather conditions influenced Strongylidae egg counts (Table 2), in contrast to the results of two studies in Etosha National Park that reported higher egg counts in the rainy than the dry season (Turner and Getz 2010; Cizauskas et al. 2015) and attributed lower infection loads in the dry season primarily to the poor survival of larvae. In contrast, studies in South Africa report higher egg counts in drier than in wetter months (Scialdo-Krecek et al. 1982; Krecek et al. 1987a). These regional differences may be influenced by variation in species composition and fecundity of the Strongylidae community given that approximately 47 species have been described to infect equids of sub-Saharan Africa (Scialdo-Krecek and Bigalke 1983; Round 1986; Matthee et al. 2004; Kuzmina et al. 2012). Moreover, cyathostomins typically occur at higher numbers in the equid intestines than large strongyles (Corning 2009; Bowman 2013; Nielsen and Reinemeyer 2018), and their larvae are more likely to encyst when high numbers of adult cyathostomins occur in the host’s large intestine (Corning 2009), a condition most likely in the Serengeti National Park during the wet season. The expected decline in infection during the dry season may thus have been masked by the emergence of encysted larvae during dry conditions.

We reasoned that environmental contamination with infective larvae should be higher when zebras occur in large aggregations which should lead to an increased likelihood of transmission. Our analyses revealed that aggregation size had no effect on nematode egg counts in adults of either sex (Tables 2 and 3), whereas young zebras in large aggregations had higher ascarid egg counts than those in small aggregations (Table 3). Aggregation size also had no effect on the occurrence of cestode infection regardless of age and sex (Table 4). Even so, we did find a non-significant trend for adult males to have higher ascarid egg counts when in larger and smaller aggregations. Our results suggest that zebra bands manage to avoid contaminated forage to some extent, possibly by not grazing areas near feces.

### Co-infections

Plains zebras with high infection loads of energetically costly nematode parasites are expected to have compromised immune processes and may thus be more susceptible to co-infections (Beldomenico et al. 2008; Beldomenico and Begon 2010; Jolles et al. 2015). In line with this prediction, our analyses revealed that as Ascarididae egg counts increased in adult females, the likelihood of co-infection with Anoplocephalidae cestodes increased (Table 3). Infection with ascarids is considered to reduce resistance to other pathogens (Bowman 2013). Ascarids are also costly parasites in terms of their damage to the host’s gut lining and consumption of host blood. Interestingly, adult females infected with ascarids were less likely to be co-infected with Anoplocephalidae cestodes and vice versa. Interpretation of results on co-infections in young plains zebra is problematic because only four individuals were infected with both Ascarididae and Anoplocephalidae. Generally, direct effects between adult Ascarididae and Anoplocephalidae are unlikely as they inhabit different regions of the intestine (Bowman 2013). Modulation of host immune responses by Ascarididae is likely (Maizels et al. 2018) and may benefit Anoplocephalidae. Energetically costly parasites such as ascarids and strongyles should compromise immune functions and lead to increased parasite burdens, but we found no evidence to support this in terms of strongyle infections. Our results suggest more complex interactions between parasite groups and their hosts, including potential sex differences in these synergies (Lee 2006; Klein and Flanagan 2016). Although plains zebra in various African countries are known to be infected by anoplocephalid cestodes (Monning 1928; Scialdo-Krecek et al. 1982; Wambwa et al. 2004; Fugazzola and Stancampiano 2012), our study is the first to report infection of plains zebra in Tanzania with these parasites. Preliminary molecular genetic results suggest that the anoplocephalid species observed in our samples is *A. perfoliata* (data not shown).

### Concluding remarks

The conventional non-invasive method of fecal egg counts provides only an approximation of parasite infection burdens; thus, results should be interpreted with caution (Dowdall et al. 2002; Kuzmina et al. 2012; Lester and Matthews 2014). Even so, this method has provided many useful insights into parasite–host interactions at the individual and ecological level (Festa-Bianchet 1989; Cattadori et al. 2005; Behnke et al. 2005). We observed no signs of severe parasitoses such as severe diarrhea, lethargy, or malnourishment in zebras with very high levels of parasite infection (> 4000 EPG of Strongylidae and > 1600 EPG of Ascarididae). Life history stage exerted stronger effects on parasite infections in adult males than adult females and young. Additional research on immunological and endocrine effects on such differences between sexes would be needed to elucidate this phenomenon. The effect of climatic conditions was less pronounced than expected, perhaps because the study was conducted in a year with above-average precipitation at the start of the dry season. Future studies should thus examine how differences between ecosystems and climatic conditions may affect intestinal parasite infections in wild equids and to elucidate the effects of gastrointestinal parasite infection on host immunity and Darwinian fitness in wild equids.

## Electronic supplementary material

ESM 1 (DOCX 1846 kb)
