# Supplementary material for: Effects of life history stage and climatic conditions on fecal egg counts in plains zebras (Equus quagga) in the Serengeti National Park
Source: Parasitol Res. 2020 Aug 11;119(10):3401–13. doi: 10.1007/s00436-020-06836-8 (PMC7505882; doi:10.1007/s00436-020-06836-8)
Supplement: Supplementary file 1 — (DOCX 1846 kb) [file 436_2020_6836_MOESM1_ESM.docx]

**
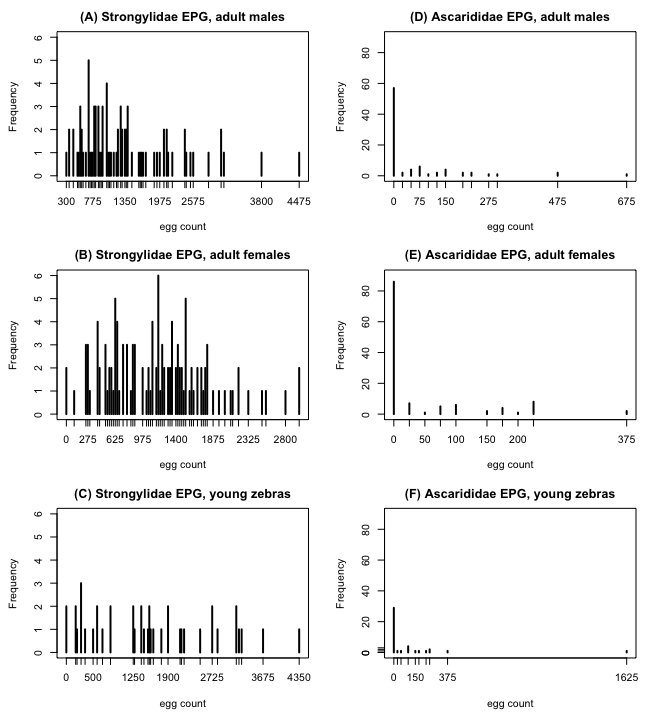
**

**Supplementary fig. 1** Frequencies of Strongylidae egg counts (EPG; panels A-C) and Ascarididae egg counts (panels D-F) in fecal samples of adult males (panels A and D), adult females (panels B and E), and young zebras (panels C and F), respectively

**Supplementary table 1** Summary of models and used R packages (R version 3.5.2)

| Parasite group | model | predictors |
| --- | --- | --- |
| Strongylidae  egg counts | generalized linear models, negative binomial distribution (package MASS version 7.3-51.4) | *-climatic condition*  *-reproductive state (adults)/age class (young)*  *-aggregation size*  *-Anoplocephalidae co-infection*  *-Ascaridae co-infection* |
| Ascaridae egg counts | generalized linear models, zero-inflated negative binomial distribution (package pscl, version 1.5.5) | *-climatic condition*  *-reproductive state (adults)/age class (young)*  *-aggregation size*  *-Anoplocephalidae co-infection* |
| Anoplocephalidae infection | binary logistic regression models (package glmmTMB, version 1.0.1) | *-climatic condition*  *-reproductive state (adults)/age class (young)*  *-aggregation size*  *-Ascaridae co-infection* |

**Supplementary table 2** Proportions of data points under dry and wet climatic conditions and in large and small aggregations

| Climate  Aggregation size | dry | wet |
| --- | --- | --- |
| large (> 200 zebras) | *24.9%* | *21.3%* |
| small (≤ 200 zebras) | *47.0%* | *6.8%* |
